# Generating Important Insights into the Spectrum and Outcomes of Acute Heart Failure Across the African Continent: The Sub-Saharan Africa Survey of Heart Failure (THESUS-HF II)

**DOI:** 10.5334/gh.1449

**Published:** 2025-07-23

**Authors:** Karen Sliwa, Simon Stewart, Charle Viljoen, Shaazia Allie, Julia Hahnle, Albertino Damasceno, Neusa Jessen, Mahmoud Sani, George Nel, Duard Smith, Beth Davison, Gad Cotter

**Affiliations:** 1 Cape Heart Institute, Department of Medicine & Cardiology, University of Cape Town, South Africa; 2 Institute of Health Research, University of Notre Dame Australia, Fremantle, WA, Australia; 3 Department of Medicine, Maputo Central Hospital, Eduardo Mondlane University, Mozambique; 4 Depart of Medicine, Division of Cardiology, Aminu Kano Teaching Hospital, Bayero University, Nigeria; 5 Pan-African Society of Cardiology, Cape Town, South Africa; 6 Momentum Research Inc. Durham, NC, United States

**Keywords:** Acute heart failure, epidemiology, prognosis, clinical profile, health economics, cardiomyopathy, Africa

## Abstract

**Background:**

Heart failure (HF) affects approximately 64.3 million people worldwide. Despite notable progress over the past two decades in advancing the understanding of heart failure in Africa–a condition often more lethal than many cancers–important knowledge gaps persist. These include outdated data on access to care and a lack of information regarding the incidence, aetiology, availability, and affordability of HF medications.

**Objectives:**

To prospectively characterise the contemporary incidence, epidemiology, clinical presentation, and health outcomes of acute HF among a large, representative cohort of patients presenting to hospitals across diverse communities in Africa.

**Methods:**

The Sub-Saharan Africa Survey of Heart Failure (THESUS-HF II) is a pragmatic, multicentre, observational cohort study coordinated by the Pan-African Society of Cardiology (PASCAR). All 27 PASCAR member countries were invited to participate, along with over 5,000 clinicians from the PASCAR database. The survey comprises two components. First, a platform collecting data on each hospital’s catchment population, human resources, presence of specialised cardiology services, availability of diagnostic tools, and access to essential heart failure treatments. Second, a prospective observational study capturing all acute heart failure presentations to participating hospitals over seven weekdays within an 8-week period (from the start of surveillance). Data were collected on clinical characteristics and outcomes to discharge, 30 days, and six months. The study commenced in mid-2024 and includes approximately 50 hospitals across 16 countries spanning all major regions of the African continent.

**Conclusions:**

When completed, THESUS-HF II will be the largest and most comprehensive study of acute HF to date in Africa. It will provide invaluable insights into the contemporary characteristics and burden of acute HF in Africa, whilst indicating what is needed to improve health care planning and, ultimately, patient outcomes.

## Introduction

Current estimates indicate that approximately 64 million people worldwide are living with–and succumbing to–heart failure (HF) (1). Therefore, it is essential to understand and address this complex syndrome, driven by multiple pathophysiological mechanisms that lead to left ventricular systolic and diastolic remodelling and dysfunction. These alterations often culminate in acute decompensation episodes and, without timely and proactive intervention, significantly increase the risk of premature mortality (2,3,4). Accordingly, significant efforts have been made to map the global burden of HF (5,6,7), in order to better understand how ‘proven’, guideline-based treatment and management modalities (2,3,4) can be effectively implemented. As previously demonstrated by multinational initiatives such as the International Congestive Heart Failure (INTER-CHF) Study (8) and the Global Congestive Heart Failure (G-CHF) Registry (9), the causes, consequences, and overall epidemiological profile of HF are highly heterogeneous. This reflects fundamental global differences in the causes of cardiac dysfunction, further compounded by inequitable access to healthcare, from basic services to specialized heart failure care and gold-standard treatments. Such heterogeneity is an important consideration when interpreting reviews of the published HF epidemiology, as these largely draw upon data from high-income regions like North America and Europe (10). Overall, there is still a paucity of data from the 85% of the world’s population living in low- to middle-income countries (LMICs) (11,12,13). Based on this limited perspective, HF is mostly reported as a condition affecting older individuals (typically aged >65 years), predominantly caused by ischemic heart disease and affecting significantly more men than women (5,6,7). It is on this basis that, historically, the diagnosis and treatment of HF have mainly focused on those presenting with an ejection fraction (EF) below the thresholds of 40% (reduced EF) and 50% (mildly reduced EF), thereby excluding many women who develop deadly and debilitating HF associated with preserved EF but compromised diastolic dysfunction (9,14,15). Such sex-based disparities in the understanding and treatment of HF are further exacerbated by aetiological, demographic, and cultural factors when considering populations outside high-income countries (HICs). This is especially true for the predominantly younger, low- to middle-income populations across the African continent (home to over 1.5 billion people) where the pattern of heart disease differs significantly (16). As initially revealed by pivotal, single centre studies (17,18), and the broader, regional comparisons generated by the THESUS HF study (28), INTER-CHF (8) and G-CHF (9) studies that included some African cohorts and focused on chronic forms of HF it is evident that in Africa, HF affects individuals of all ages and both genders equally. Moreover, HF in Africa has many different aetiologies. Driven by several key factors–including a higher burden of communicable diseases, exposure to indoor pollutants, widespread poverty, malnutrition, and limited access to healthcare systems (16,19,20,21,22), the pattern of HF in Africa appears to be more diverse than what is reported by the Global North (5,6,7). There is a notable paucity of data on heart failure treatment across Africa, particularly regarding the initiation, optimisation, and adherence to guideline-directed therapy (23,24,25,26,27,28,29,30). This highlights the urgent need for comprehensive, region-specific research to better understand and address the unique challenges of HF management in Africa.

## Rationale for the current study

Prompted by the paucity of data from Africa, the Sub-Saharan African Survey of Heart Failure (THESUS-HF) was initiated. It subsequently became the first and largest multicentre registry of acute HF on the continent, characterizing the causes and short-term outcomes of over 1,000 patients managed across 12 cardiology centres in nine countries (31,32,33,34,35). One of the most notable findings from the original THESUS-HF study, first published in 2012, was the relatively young age (i.e., mean age of 52 years) of the patient cohort (36). Since the data collection period of the original THESUS-HF study (2008–2010) (36), the pattern of acute heart failure across Africa’s diverse populations has likely evolved–driven by the ongoing burden of communicable diseases with cardiac implications, rapid urbanisation, shifting environmental conditions, and increasing adoption of Western lifestyle behaviours (16,21,22). While these factors are likely to exacerbate the causes and consequences of HF, guideline-directed medical therapy (GDMT) has been effectively adapted based on new evidence (37,38,39). There is also a paucity of data on the availability and affordability of heart failure therapies in Africa–particularly regarding the initiation, optimisation, and adherence to guideline-directed medical therapy (GDMT) (23,24,25,26,27,28,29,30). This gap has significant implications for patient management and healthcare planning, and has served as a key rationale for undertaking this study. In this context, we present the initial report from the Sub-Saharan Africa Survey of Heart Failure (THESUS-HF II) study, which aims to address these knowledge gaps and provide much-needed insight into heart failure across the African continent.

## Aims and Objectives

The primary aim of this pragmatic, multicentre, prospective observational study (i.e., THESUS-HF II) is to comprehensively characterize acute heart failure cases from an individual to healthcare perspective, with a unique focus on Africa. To achieve this aim, the primary study objectives are to prospectively describe the incidence, epidemiology, clinical characteristics, and outcomes (up to discharge, 30 days, and six months after the index admission) among a large, representative cohort of patients presenting acutely to hospitals with heart failure from a diverse range of communities across the African continent.

### Secondary aims and objectives

Among a range of secondary objectives, a key secondary outcome is to describe the centre-specific diagnostic and therapeutic processes applied to those presenting with acute HF across Africa. This includes evaluating how contemporary guidelines and recommendations for clinical management are applied, with a focus on potential variations due to geographic differences and seasonal factors. Information will be collected on the reasons for not prescribing evidence-based HF treatment or for prescribing HF medication at doses lower than those recommended by contemporary guidelines. Additionally, we intend to explore and identify potential diagnostic and prognostic markers that might be useful for identification and risk stratification of HF cases in Africa. Seasonal variation and regional differences in treatment patterns, outcomes, and healthcare access will also be explored to assess their impact on HF management. Furthermore, the data collected as part of THESUS-HF II will be used to provide feedback to participating sites, facilitating the improvement of patient care with the resources currently available. In broader terms, there will be a focus on deriving estimates of healthcare costs associated with acute HF, as well as addressing the broader challenges of conducting clinical research within the uniquely diverse African context.

## Methods

### Study context

As reported by the World Bank (latest population estimates available for 2023) (40) and other agencies, the African continent is home to over 1.5 billion people living across a wide spectrum of geopolitical, cultural, socioeconomic, and environmental contexts. These diverse settings profoundly influence the risk factors, presentation, and burden of heart failure and other forms of heart disease endemic to the region (16,41). The United Nations Statistics Division (UNSD) identifies five distinct regions across the African continent. Northern Africa, dominated geographically by the Sahara Desert, consists of seven countries with a combined population exceeding 250 million as of 2023/24. The remaining regions, which collectively referred to as sub-Saharan Africa, include Eastern Africa (16 countries with > 500 million people), Central Africa (9 countries with > 200 million people), Western Africa (19 countries with > 500 million people), and Southern Africa (5 countries with a population > 70 million). Each of these regions–and the individual countries they comprise–exhibit a diverse array of socio-economic and health-related factors that are likely to influence the observed patterns of HF within their respective adult populations. These factors include national economic status (as measured by gross domestic product [GDP] per capita) (42), life expectancy (43), rates of rural-to-urban migration and employment patterns (44), prevalence of obesity (45), and dietary habits, including average daily salt intake (46).

### Study design

**Figure 1:**
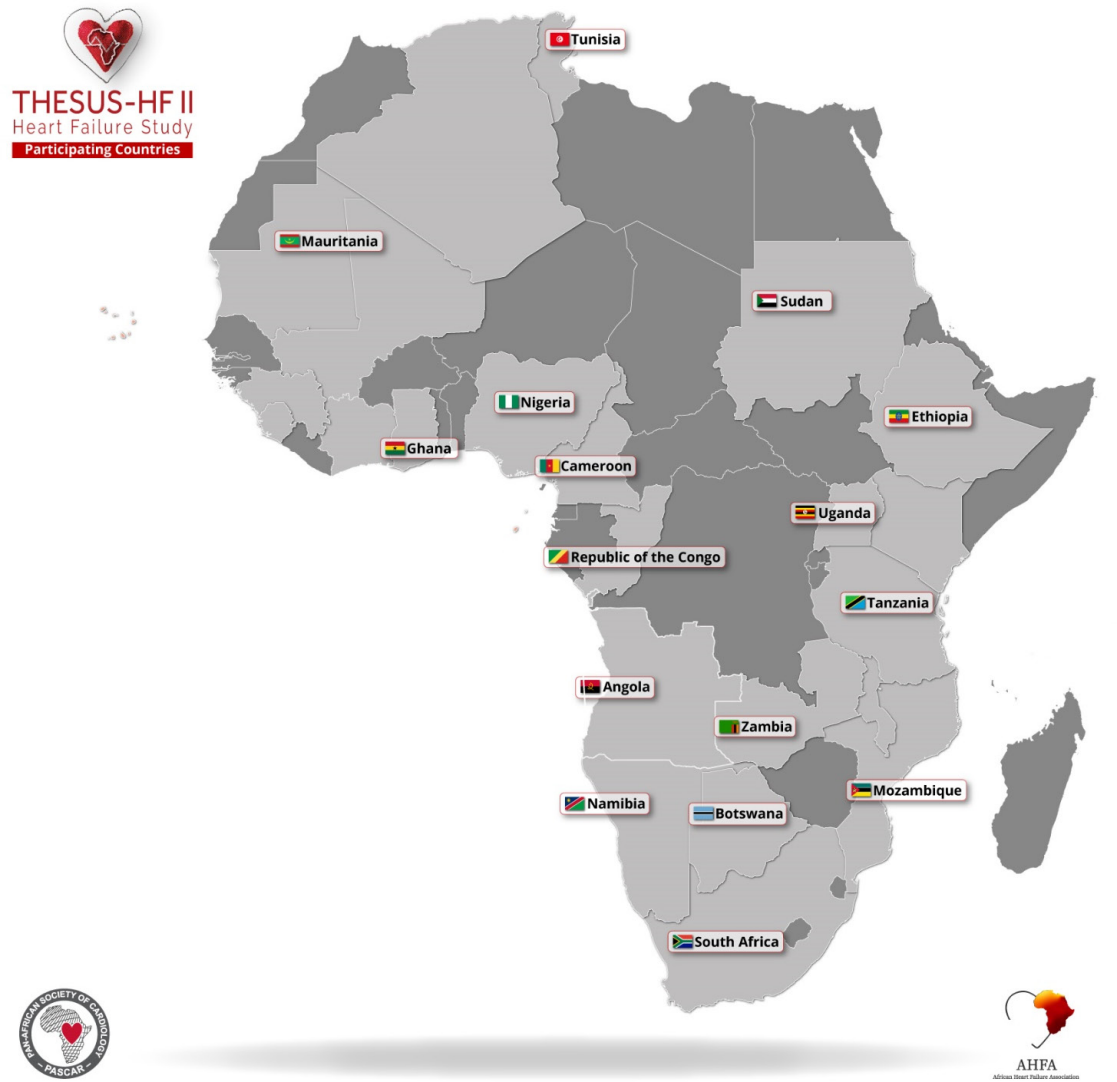
Overall distribution participating THESUS-II surveillance sites.

THESUS-HF II is a prospective, multicentre, multi-country clinical surveillance study of acute HF presentations throughout Africa (Figure 1). It is led and coordinated by the pre-eminent scientific organization dedicated to cardiac care and outcomes in Africa – the Pan African Society of Cardiology (PASCAR – https://www.pascar.org/). A robust governance structure/processes coordinated by the PI (KS) and highly experienced co-investigators team (named co-authors) has been applied.

The study adheres to the ethical principles outlined in the Declaration of Helsinki, complies with Good Clinical Practice (GCP) standards in research, and follows the latest Guidelines for Accurate and Transparent Health Estimates Reporting (GATHER) (47). Primary ethical approval (including provision for obtaining informed consent for study participants) was obtained from the Human Ethics Research Committee (HREC) at the Faculty of Health Sciences (FHS) of the University of Cape Town (UCT), South Africa (UCT FHS HREC ref no 897/2023). Following this approval, local ethical approval was obtained from the relevant ethics committees at each participating centre, as appropriate (listed in **Appendix I**).

### Study sites

Through PASCAR, all 27 member societies were contacted and invited to participate in the study. Recognizing that some countries lack a sufficient number of cardiologists to establish formal societies, all healthcare professionals listed in the PASCAR database (i.e., over 5,000 individuals) were also invited to contribute. This inclusive, pragmatic approach has previously proven effective in conducting large-scale disease surveillance in low-income settings, including a successful study in Mozambique (48).

Whenever a site cannot be recruited to take part in the study, the underlying reasons are investigated. Accordingly, ethical approval was sought for the THESUS-II study to explore the barriers and enablers to conducting research in Africa, with the goal of developing strategies to build sustainable health research capacity in LMICs. This approach reflects a broader commitment not only to understanding the clinical and logistical aspects of acute HF management in Africa but also to addressing the systemic challenges that hinder equitable research participation across the continent. By identifying factors that limit or facilitate site inclusion–ranging from ethical review processes to infrastructural and administrative capacity–we aim to inform long-term improvements in research capability, regulatory harmonization, and patient care in resource-constrained settings. These findings are planned to be discussed in a dedicated paper on the barriers and enablers of conducting HF research in Africa.

### Country and site information

Prior to the formal initiation of the study, each participating site was required to complete a comprehensive site information sheet as part of the participation agreement. This form gathered essential data on hospital size and type, the demographic profile of the population served, the number and specialisation of treating physicians, and the availability and cost of medications, medical devices, and cardiac catheterization facilities. These details will help define the local context in which patients with acute heart failure are managed.

A key requirement for participation in THESUS-HF II is the ability to provide data on the catchment population served by each referral hospital, as well as the capacity to perform essential diagnostic investigations, namely electrocardiography (ECG), chest radiography (CXR), and point-of-care (POC) echocardiography (echo)–at the treating facility (see below).

### Study surveillance

In alignment with the practical challenges of conducting this project in numerous low-resource settings across the African continent, and drawing on the approach successfully used in a prior multicentre disease surveillance project in Mozambique (48), each participating site is tasked with collecting data on consecutive acute HF cases over seven 24-hour periods within an 8-week window (starting from the first day of surveillance). Each 24-hour period must fall on a different day of the week to ensure representative case capture.

### Study cohort

Each participating site records the total number of adult medical cases (aged 18 years and older) presenting to the hospital emergency department during each 24-hour surveillance period, including suspected acute HF cases. Potential acute HF cases are then identified and evaluated based on symptoms such as shortness of breath or oedema, along with clinical signs like peripheral oedema, raised jugular venous pressure, and/or pulmonary crackles.

### Participant profiling data

After obtaining informed consent, study data are collected on standardized case-report forms (CRFs) as soon as practicable. Since the study is observational, participation does not impact clinical management. Standard management of patients, including diagnostic and therapeutic interventions currently performed at each centre for patients presenting with signs and symptoms of HF, is recorded. Study participants are enrolled only if they have undergone an ECG, chest X-ray (CXR), and echocardiogram during their hospital admission. These mandated investigations are performed as soon as possible but prior to discharge. The participant’s NT-proBNP level is also recorded if laboratory testing is available at the site and measured as part of routine care. Drug prescriptions, dosages, and decisions to perform other diagnostic or therapeutic procedures are left to the discretion of the participating physician. No specific protocols or recommendations for evaluation, management, or treatment are provided during this observational study.

Standardized data collection also includes each participant’s demographic characteristics, cardiovascular risk factors, family history of cardiomyopathy, comorbidities, clinical signs and symptoms, and blood test results.

Mandatory echocardiographic parameters include LV dimensions and LV ejection fraction (LVEF), left atrial size, presence of rheumatic or other valvular disease, pericardial effusion, and visible thrombus. Mandatory ECG parameters include rate, rhythm, PR interval, QRS duration, QT interval, presence of left ventricular hypertrophy (LVH), and T wave inversion. Detailed information on the pharmacological and non-pharmacological therapies applied to each participant is also collected.

### Health outcomes

Outcome data include each participant’s length of hospital stay, admission to an Intensive Care Unit (ICU), and survival status (alive or deceased) at hospital discharge. Additionally, for those who survive the index hospitalization, the study aims to collect data on 30-day to 6-month survival and hospital readmission status through a brief telephone survey whenever possible. These data, combined with site profiling and participant information, will assist in informing health economic analyses of the cost burden of acute heart failure from site- and region-specific perspectives to an overall pan-African view.

### Data management

All study site and participant data are recorded through an online electronic case report form (CRF) using a secure, password-protected Research Electronic Data Capture (REDCap) system, which is maintained by the Cape Heart Institute, University of Cape Town, South Africa. Each participant receives a unique study ID number to ensure anonymity during data transfer. The central data management team applies standard protocols to verify source data and ensure the quality of the data reported.

## Statistical analyses

All analyses will be conducted according to a pre-specified statistical analysis plan. As a pragmatic, observational study, no formal power calculations have been conducted. Continuous variables will be presented as means with standard deviations (SD) for parametric data or as medians with interquartile ranges (IQR) for non-parametric data and will be compared using the Student’s t-test, Wilcoxon rank-sum test, or Mann-Whitney U test, as appropriate. Categorical variables will be reported as frequencies and percentages and will be compared using the chi-squared test or Fisher’s exact test. Kaplan-Meier survival plots will be used to illustrate time to death or hospital readmission following the index admission for the entire cohort. Comparisons across UNSD regions will be performed based on patient characteristics and outcomes, including weekday versus weekend timing. Multivariable regression models will be employed to assess associations between key exposures and heart failure outcomes, with adjustment for potential confounders. These include age, sex, socioeconomic status, comorbidities (such as hypertension, diabetes, and chronic kidney disease), aetiology, clinical severity at presentation, and access to diagnostic or therapeutic interventions. Covariate selection will be guided by clinical relevance and univariable analyses. Where appropriate, models will be stratified by region or facility type to account for site-level heterogeneity. A two-sided p value of < 0.05 will be interpreted as statistically significant.

The indicative incidence of acute HF (from a UNSD regional to pan-African perspective, the former being dependent on the number of cases captured as part of the study) will be calculated from – a) the total population aged 18 years or more at risk (derived from the total populations being serviced by participating hospitals (*denominator*) and b) the total number of acute HF cases (*numerator*) documented over the 7-day observation periods (*time exposure*). The incidence of acute HF will be expressed as the number of cases/10,000 population at risk per week, with extrapolation to an annual estimated caseload of acute HF overall, on an age- and sex-specific basis and for each UNSD region represented in the study. Sensitivity analyses around these incidence estimates will be generated according to – a) high and low-estimates of the population at risk and b) the 95% CI for observed counts (of acute HF cases). Although not definitive, these methods mirror those used to generate indicative incidence of valvular heart disease in the urban enclave of Soweto in South Africa (49).

### Sub-analyses

To investigate potential **regional differences** in HF presentation, management, and outcomes, data will be categorized based on geographical regions within Africa (e.g., West, East, Southern, and Central Africa). This classification will consider regional variations in healthcare infrastructure, access to treatment, and socioeconomic factors. Comparative analyses will be performed using statistical methods such as chi-square tests for categorical variables and ANOVA or Kruskal-Wallis tests for continuous variables to identify any regional disparities in HF care and outcomes across different African settings.

To explore potential **seasonal variation** in HF presentations, management, and outcomes, data collection periods will be grouped according to meteorological seasons relevant to each study region (e.g., rainy vs. dry seasons or summer vs. winter, depending on local climate patterns) (21). Patient characteristics, treatment patterns, and in-hospital outcomes will be compared across these seasonal groupings using descriptive statistics and appropriate inferential analyses. This approach will allow assessment of whether seasonality influences HF burden and care delivery.

## Results

As summarised in Table 1, the project’s prospective surveillance protocol is expected to generate data from a total of 16 African countries (representing a combined population of 813.1 million people) that have agreed to participate in this study and were able to proceed accordingly (Figure 1). Despite the inability of many centres and investigators who were willing but unable to participate, these countries reflect the inherent diversity in ethnic, cultural, behavioural, and socio-demographic profiles of adult populations at risk of developing HF across all major regions of Africa. As outlined in the Methods section, data will also be collected to systematically identify the reasons and barriers that impeded participation by countries and sites that initially expressed willingness to join the study.

**Table 1:** Socio-economic and health indicators characteristics of THESUS-II represented countries.


REGION/COUNTRY	TOTAL POPULATION	AGED 15–64 YEARS	AGED 65+ YEARS	GDP PER CAPITA (USD)/STATUS	LONGEVITY (YEARS)	URBAN-DWELLING	ADULT OBESITY M/F	NA INTAKE (g/DAY)

**NORTHERN/SAHARAN AFRICA**								

Tunisia	12.46 M	60%	6%	$3,895/LMI	74.3	20%	19.9%/35.3%	6.10

Mauritania	5.17 M	56%	3%	$2,149/LMI	65.7	58%	9.3%/36.2%	2.80

**WESTERN AFRICA**								

Ghana	32.83 M	55%	3%	$2,363/LMI	63.8	59%	5.4%/21.1%	2.35

Nigeria	213.4 M	54%	2%	$2,066/LMI	52.7	54%	8.5%/17.3%	2.82

**CENTRAL AFRICA**								

Angola	34.50 M	53%	3%	$1,954/LMI	61.6	68%	6.5%/16.9%	2.49

Cameroon	27.20 M	53%	3%	$1,667/LMI	60.3	59%	9.7%/21.2%	2.09

Congo (Dem. Rep.)	95.89 M	51%	3%	$577/LI	59.2	47%	4.4%/9.4%	2.42

**EASTERN AFRICA**								

Ethiopia	120.3 M	58%	3%	$925/LI	65.0	23%	1.1%/4.7%	2.27

Mozambique	32.08 M	54%	3%	$492/LI	59.3	38%	6.5%/14.3%	2.24

Sudan	45.66 M	54%	2%	$752/LI	65.3	36%	11.7%/23.2%	2.37

Tanzania	63.59 M	54%	2%	$1,099/LMI	66.2	37%	6.8%/19.0%	2.75

Uganda	45.85 M	54%	2%	$884/LI	62.7	26%	4.3%/11.8%	2.11

Zambia	19.47 M	54%	2%	$1,137/LMI	61.2	46%	5.3%/17.0%	2.27

**SOUTHERN AFRICA**								

Botswana	2.592 M	64%	4%	$6,805/UMI	61.1	72%	8.8%/28.3%	2.53

Namibia	2.530 M	60%	4%	$4,866/LMI	59.3	54%	10.7%/23.8%	2.64

South Africa	59.39 M	60%	6%	$7,055/UMI	62.3	68%	14.5%/47.3%	2.48


**Legend:** M = million, LMI = low-to-middle income country, LI = low-income country. Specific head-to-head demographic, socio-economic and health indicators were derived from – 1) https://databank.worldbank.org/source/population-estimates-and-projections 2) https://data.worldobesity.org/rankings/?age=a&sex=t, and 3) https://worldpopulationreview.com/country-rankings/salt-consumption-by-country.

**Figure 2:**
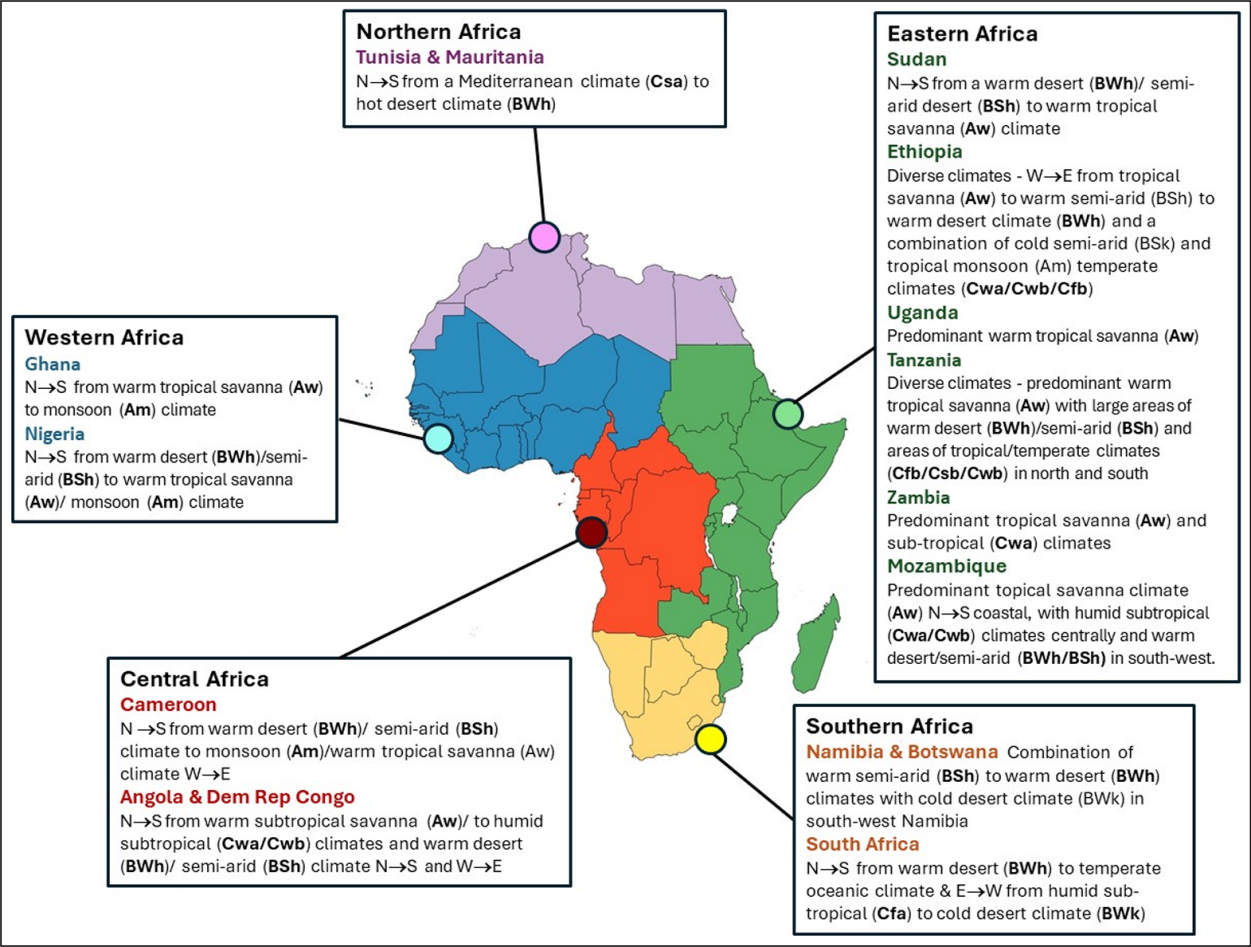
Geographic and climate distribution of THESUS-II surveillance sites. **Legend:** Climate profiling is based on the Köppen climate classification system (50).

As illustrated in Figure 2, geographic diversity also encompasses external and climatic factors that may precipitate acute cardiac decompensation, such as heatwaves and other extreme weather conditions. Based on the current progress of the project, it is anticipated that by June 2025, participating surveillance sites (listed in **Appendix I**–participating sites and site investigators) will have enrolled over 1,000 patients presenting with acute heart failure across all five major UNDA regions of Africa.

## Discussion

To our knowledge, building on the original THESUS-HF study (36), involving 9 countries and 1,006 patients, the expanded THESUS-HF II project represents the largest and most representative study of acute heart failure presentations in Africa to date. While it does not fully realize the potential scope of such research–underscoring the critical need for increased capacity building and funding (16)–THESUS-HF II, upon completion by the end of 2025, will provide detailed country-, centre-, and patient-specific data from at least 16 countries across all major UNSD regions of the diverse African continent. The study will generate a range of contemporary findings related to the characteristics, incidence, treatment, healthcare availability and associated costs, as well as health outcomes of acute heart failure in Africa. In doing so, it will complement and expand upon the original THESUS-HF Study, offering valuable insights into how the epidemiology and burden of heart failure have evolved across the continent over the past decade and beyond.

### Capacity building

Reflecting the comprehensive scope of the project, THESUS-HF II incorporates an integrated Capacity Development Program. As part of this initiative, all Study Investigators will be invited to attend an annual two-day meeting–either in-person or hybrid–focused on research methodology and heart failure management specific to Africa. The format and extent of these meetings will depend on the funding secured. Recognizing that surveys and cohort studies are essential tools for understanding disease natural history and associated risk factors, the sessions will cover key topics including the formulation of research questions, cohort study design, Good Clinical Practice (GCP) and regulatory considerations, as well as data collection, analysis, and interpretation. From our recent publication of the STRONG-HF study (37), we have learned that rapid up-titration of heart failure therapies under close follow-up (including clinical examination and NT-proBNP monitoring) is safe, reduces heart failure readmissions and all-cause mortality, and improves patients’ health-related quality of life. The annual investigators’ meeting will aim to promptly disseminate these findings. Educational sessions will focus on implementing the STRONG-HF protocol into routine clinical practice, thereby facilitating guideline-directed heart failure management within low-resource settings.

### Heart Failure Advocacy Program

As part of the project, contingent upon successful funding, we plan to collaborate with the Investigators to develop a simple Heart Failure Patient Advocacy training program tailored for nurses within their specific healthcare settings. The program’s design will be informed by data collected through the survey, such as the number of newly diagnosed HF patients per site, patients’ educational levels, their current understanding of HF symptoms and medications, and issues related to medication affordability and access to care. The resulting program will incorporate the ‘patient’s voice’ and may include short videos, straightforward educational materials, and/or an online platform to effectively engage and empower both nurses and patients.

### Limitations

We acknowledge that the mandatory requirement for ECG, CXR, and echocardiography for patient inclusion (although essential for accurate diagnosis), may introduce selection bias in settings where these diagnostic tools are not readily accessible. Consequently, patients without access to these investigations are excluded from the study, potentially limiting the generalizability of our findings to resource-constrained environments lacking such capacity. Additionally, the study relies on point-of-care echocardiography performed at participating sites, which may be subject to inter-observer variability. In the absence of a central quality control or standardized adjudication process, the accuracy and consistency of echocardiographic data will depend heavily on the expertise of local operators. This variability may impact data uniformity across sites and is recognized as a limitation of the study.

We also acknowledge that varying referral patterns and differences in healthcare access across settings may introduce bias and limit the accuracy of HF incidence estimates. While we hope to derive meaningful HF incidence data, we recognize these challenges and will address this limitation explicitly, where appropriate. Additionally, while 30-day and 6-month follow-ups conducted via telephone are practical and feasible in diverse, resource-limited environments, they carry risks of loss to follow-up and difficulties in verifying mortality. Nonetheless, this method represents the most viable approach for longitudinal data collection given the logistical and infrastructural constraints at participating sites. We will encourage all efforts to maximize patient contact and outcome verification, transparently report follow-up rates, and consider these limitations in our analysis and interpretation of results.

## Conclusions

Building on insights from the original THESUS-HF (31,32,33,34,35), THESUS-HF II aims to provide contemporary, comprehensive data on the clinical presentation and management of acute heart failure across Africa. This effort is timely given the rising burden of heart failure on the continent, which is marked by distinct differences in clinical characteristics, causes, management, and outcomes compared to high-income countries (10). This increasing burden is driven by epidemiological transitions, a growing prevalence of non-communicable disease risk factors, urbanization, lifestyle changes, and ongoing challenges such as infectious diseases and healthcare disparities. By capturing real-world data on risk factors, aetiology, treatment practices, and short- to medium-term outcomes from a large, diverse cohort, the study aims to support evidence-based healthcare planning and optimize resource allocation tailored to Africa’s unique healthcare context.

Importantly, the findings from THESUS-HF II will not only inform clinical practice but also serve as a powerful advocacy tool to engage policymakers, health ministries, and regional stakeholders by identifying systemic gaps in diagnostic capacity, treatment availability, and patient follow-up infrastructure. This will lay the groundwork for targeted interventions and national strategies to improve heart failure care, accelerating the integration of affordable, contextually appropriate management guidelines into national health policies and training programs. Additionally, the lessons learned (particularly regarding logistical and regulatory challenges in conducting large-scale, multi-country research) will guide future efforts to establish sustainable research networks across Africa. Thus, the project contributes both to immediate improvements in patient care and to strengthening the continent’s long-term capacity for impactful, policy-relevant clinical research.

## Additional File

The additional file for this article can be found as follows:

10.5334/gh.1449.s1 Appendix I. Participating sites and investigators.
