# Supplementary material for: Generating Important Insights into the Spectrum and Outcomes of Acute Heart Failure Across the African Continent: The Sub-Saharan Africa Survey of Heart Failure (THESUS-HF II)
Source: Glob Heart. 2025 Jul 23;20(1):64. doi: 10.5334/gh.1449 (PMC12292052; doi:10.5334/gh.1449)
Supplement: Appendix I. — Participating sites and investigators. [file gh-20-1-1449-s1.pdf]

PARTICIPATING SITE INVESTIGATORS AND RESEARCH TEAMS

Luanda, Angola, Angola Military Hospital: F. Nataniel, L. Janetis, V. Selombo, **Gaborone, Botswana**, Princess Marina Hospital: J. Mwita, M. Goepang, **Buea, Cameroon**, Buea Regional Hospital: C. Nkoke, **Douala, Cameroon**, Hospital General de Douala: A. Dzudie, S. Umaru, **Adama, Ethiopia,** Adama Hospital Medical College: D. Tesfaye, Y. Bekele, **Dessie, Ethiopia,** Dessie Comprehensive Specialized Hospital: S.G. Abdela, A. Yimer, T. Kumie, **Hawassa, Ethiopia,** Hawassa University Comprehensive Specialized Hospital: S.M. Gobu, K. Woubshet, B. Bikamo, **Addis Ababa, Ethiopia,** Tikur Anbessa Specialized Hospital: T. Mekonnen, D. Yadeta, C. Fekadu, **Addis Ababa, Ethiopia,** ICMC General Hospital: M. Alemayehu, Amsalu, Abel, **Addis Ababa, Ethiopia**, Lancet General Hospital: D. Mekonnen, Z.D. Jemaneh, Y. Awoke, **Kumasi, Ghana,** Komfo Anokye Teaching Hospital: L. Appiah, Y. Adu-Boakye, C. Kokuro, **Nouakchott, Mauritania,** Centre National de Cardiologie: B. Fatimata, S. Boubacar, M.M. Walata, **Maputo, Mozambique,** Maputo Central Hospital: A. Damasceno, N. Jessen, I. Mbanze, **Windhoek, Namibia,** Windhoek Central Hospital: T. Auala, N.W. Agapitus, **Sagamu, Nigeria,** Olabisi Onabanjo University Teaching Hospital: F. Inofomoh, O.B. Familoni, A. Temitope, **Dutse, Nigeria,** Rasheed Shekoni Federal University Teaching Hospital: S. Ringim, S. Mohammed Ha, U.A. Abdullah, **Kano, Nigeria**, Aminu Kano Teaching Hospital: M. Sani, S.A. Kana, T. Abdussalam, **Enugu, Nigeria**, Enugu State University of Science & Technology Teaching Hospital: O. Okeckukwu, C. Faith, U. Afam, **Oghara, Nigeria,** Delta State University Teaching Hospital: E. Umuerri, **Keffi, Nigeria,** Federal Medical Centre Keffi: V. Shidali, C. Imo, **Gombe, Nigeria,** Federal Teaching Hospital Gombe: O. H Ifeanyichukwu, Y. A. Ayoola, A. I. Olayemi, **Jos, Nigeria,** Jos University Teaching Hospital: G. Amusa, **Ikeja, Nigeria,** Lagos State University Teaching Hospital: F. Daniel, P. Adebola, R. Moronkola, **Kano, Nigeria,** Murtala Muhammad Specialist Hospital: B.G. Ahmad, Y.S. Umar, H. Saidu, **Ile Ife, Nigeria,** Obafemi Awolowo University Teaching Hospitals Complex: R. Adebayo, A.O. Akintomide, O.A. Akinyele, **Ogbomoso, Nigeria,** LAUTECH Teaching Hospital : A. Akintunde, O. Opeyemi, **Lagos, Nigeria,** Lagos Executive Cardiovascular Centre Lagos: F. Alli, A. Yaguda, G. Udu**, Lagos, Nigeria,** Lagos University Teaching Hospital: A. Mbakwem, C. Amadi, O. Ale, **Port Harcourt, Nigeria,** Rivers State University Teaching Hospital: C. Alikor, B. Oyan, E. Nyeche, **Osogbo, Nigeria**, Uniosun Teaching Hospital: P. Akinwusi, J. Olarewaju, O.L. Adebiyi, **Ibadan, Nigeria,** University College Hospital: O. Ogah, O. Oladapo, **Calabar, Nigeria,** University of Calabar Teaching Hospital: V. Ansa, I. Ukpeh, E. Epoke, **Maiduguri, Nigeria,** University of Maiduguri Teaching Hospital: M.A. Talle, F. Buba, I.A. Galtimari, **Portharcourt , Nigeria,** University of Port-harcourt Teaching Hospital: A. Ajala, M. Akpa, S. Dodiyi-Manuel, **Gombe, Nigeria,** State Specialist Hospital : O. H. Ifeanyichukwu, H. Abubakar, **Uyo, Nigeria,** University of Uyo Teaching Hospital: T. Shogade, A.U. Udosen, E.M. Udoh, **Abuja, Nigeria,** College of Health Sciences University of Abuja: D. Ojjji; **Brazzaville, Republic of the Congo**, University Hospital of Brazzaville: B.F.E. Mbolla, C.M.K. Landa, R.P. Bakekolo, **Sao Tome, São Tomé and Príncipe,** Hospital Ayres de Menezes: M. Cassandra, **Cape Town, South Africa,** False Bay Hospital: J. Porter, **Khartoum, Sudan,** Alshaab Teaching hospital: M. Mohammed, M. Bashir, **Shendi, Sudan,** Elmek Nimir University: M. Babker, A. Alhassan, A. Azhari, Khartoum, **Sudan, Alshaab** Teaching Hospital EC: m. Mohammed, M. Bashir, **Mwanza, Tanzania,** Bugando Medical Centre: F. Kalokola, D. Mabula, **Dar es salaam, Tanzania**, Centre of Excellency Cardiovascular Sciences Mloganzila Hospital: C. Kabakama, R. Minja, A. Valentino, **Tunis, Tunisia,** Security Forces Hospital: L. Zakhama, S. Antit, E. Boussabah, **Kampala, Uganda,** Kiruddu National Referral Hospital: C. Mondo, O. Jane, N. Stella, **Lusaka, Zambia,** National Heart Hospital: L. Kabwe, D. Chibomba, S. Essa, **Lusaka, Zambia,** Levy Medical University Teaching Hospital: J. Musuku, F. Yavwa, D. Chimanuka, **Kitwe, Zambia,** Kitwe Teaching Hospital: B. Chanda, J. Mubita, S. Mushota
